# *Artemisia* spp. essential oils against the disease-carrying blowfly *Calliphora vomitoria*

**DOI:** 10.1186/s13071-017-2006-y

**Published:** 2017-02-13

**Authors:** Stefano Bedini, Guido Flamini, Francesca Cosci, Roberta Ascrizzi, Maria Cristina Echeverria, Lucia Guidi, Marco Landi, Andrea Lucchi, Barbara Conti

**Affiliations:** 1 Department of Agriculture, Food and Environment, University of Pisa, Via del Borghetto 80, 56124 Pisa, Italy; 2 Department of Pharmacy, University of Pisa, Via Bonanno 6, 56126 Pisa, Italy; 3 Universidad Tecnica del Norte, v 17 de Julio 5-21, Ibarra, Ecuador

**Keywords:** Botanical insecticides, Repellent, Blowflies, Acetylcholinesterase, Bactericidal, Fungicidal

## Abstract

**Background:**

Synanthropic flies play a considerable role in the transmission of pathogenic and non-pathogenic microorganisms. In this work, the essential oil (EO) of two aromatic plants, *Artemisia annua* and *Artemisia* *dracunculus*, were evaluated for their abilities to control the blowfly *Calliphora vomitoria. Artemisia annua* and *A. dracunculus* EOs were extracted, analysed and tested in laboratory bioassays. Besides, the physiology of EOs toxicity and the EOs antibacterial and antifungal properties were evaluated.

**Results:**

Both *Artemisia* EOs deterred *C. vomitoria* oviposition on fresh beef meat. At 0.05 μl cm^-2^ *A. dracunculus* EO completely inhibited *C. vomitoria* oviposition. Toxicity tests, by contact, showed LD_50_ of 0.49 and 0.79 μl EO per fly for *A. dracunculus* and *A. annua*, respectively. By fumigation, LC_50_ values were 49.55 and 88.09 μl l^-1^ air for *A. dracunculus* and *A. annua*, respectively. EOs AChE inhibition in *C. vomitoria* (IC_50_ = 202.6 and 472.4 mg l^-1^, respectively, for *A. dracunculus* and *A. annua*) indicated that insect neural sites are targeted by the EOs toxicity. Finally, the antibacterial and antifungal activities of the two *Artemisia* EOs may assist in the reduction of transmission of microbial infections/contaminations.

**Conclusions:**

Results suggest that *Artemisia* EOs could be of use in the control of *C. vomitoria*, a common vector of pathogenic microorganisms and agent of human and animal cutaneous myiasis. The prevention of pathogenic and parasitic infections is a priority for human and animal health. The *Artemisia* EOs could represent an eco-friendly, low-cost alternative to synthetic repellents and insecticides to fight synanthropic disease-carrying blowflies.

## Background

Blowflies (Diptera: Calliphoridae) are problematic pests, important vectors of many foodborne, human, and domestic animal pathogens [1–4]. Feeding in animal and human excrement, garbage, and decaying organic matter, blowflies can spread microorganisms through direct contamination of food and surfaces through fecal deposits, and extracorporeal digestion (fly spots) [5, 6] causing the spread of foodborne illnesses and other diseases. In fact, blowflies have been showed to transport a variety of bacteria, cestodes, protozoans and viruses of public health importance such as *Salmonella typhimurium* [7], *Taenia* spp., *Entamoeba coli*, *Giardia duodenalis* [8], *Mycobacterium avium paratuberculosis* [9] as well as the avian influenza virus [10]. Blowflies are also characterized by the ability of their larvae to develop in the tissues of vertebrates causing myiasis, a worldwide severe medical and veterinary problem [11–13].

The bluebottle fly *Calliphora vomitoria* (L.) is a common blowfly frequently recorded in synanthropic and natural ecosystems in most areas of the world [14], and is a vector of pathogenic microorganisms [5]. Moreover, *C. vomitoria* maggots have been recorded in human and animal cutaneous myiasis [15, 16].

The prevention of blowfly infestations has traditionally relied on synthetic insecticides such as organochlorines, organophosphates and insect growth regulators [17, 18]. However, the repeated insurgence of blowfly resistance to chemicals [19] and, the issues around the harmful effects of synthetic compounds on humans [20, 21], animals [22] and the environment [23], have made new eco-friendly, low-cost tools a high priority. In this regard, essential oils (EOs) of aromatic plants, which are often characterized by low toxicity towards mammalians [24] and high biodegradability, have recently received increased attention as natural products effective as contact and fumigant insecticides and as repellents against insect pests [25–28].

*Artemisia annua* L. and *Artemisia dracunculus* L. (Asteraceae) are aromatic plants whose EOs are known for their antibacterial, antifungal and insecticidal properties [29, 30]. This study aimed to assess the toxic and oviposition deterrent activity of *A. annua* and *A. dracunculus* EOs against *C. vomitoria.* For that purpose, *A. annua* and *A. dracunculus* EOs were extracted, chemically analysed and tested in laboratory bioassays against *C. vomitoria*. The physiological mechanisms of EOs insect toxicity were evaluated by enzymatic inhibition tests. Moreover, in consideration that blowflies are vectors of pathogens, the antibacterial and antifungal properties of *A. annua* and *A. dracunculus* EOs were also evaluated against *Escherichia coli*, *Bacillus subtilis*, *Streptococcus aureus* and *Candida albicans* which are considered among the most common and harmful microbial species in mammals.

## Methods

### Flies rearing

Larvae of the bluebottle fly *C. vomitoria* were purchased from a commercial supplier (Fish Company Arco Sport, Cascina PI, Italy). The larvae were fed with beef liver and maintained under laboratory condition (23 °C, 60–70% R.H., natural photoperiod) until pupation. Species identification of the emerged adults was confirmed by a dipterologist (Prof. Alfio Raspi, Department of Agriculture, Food and Environment, University of Pisa). After identification, 20 flies were placed in a 27 × 27 × 27 cm cage, provided with solid diet (sugar and yeast 1:1) and water *ad libitum*. The sugar-yeast diet was previously shown to be successful in providing protein amounts necessary to stimulate oviposition of Calliphoridae [31, 32]. For the oviposition, beef liver was provided to females. Newly emerged larvae were fed on beef liver as well until pupation. The resulting adult *C. vomitoria* population was maintained under laboratory conditions.

### Plant material

The flowering aerial parts of *A. annua* were collected in Pisa (Italy) at the end of September 2015 along the Arno riverbanks. Aerial parts of *A. dracunculus* were collected in June 2015, during the flowering period, near Urbino, (Italy), at 500 m above sea level. The plant material was dried at room temperature in the shadow until constant weight.

### EO extraction and chemical characterisation

*A. annua* and *A. dracunculus* aerial parts were hydrodistilled in a Clevenger-type apparatus for 2 h. Gas chromatography-electron impact mass spectroscopy (GC-EIMS) analyses were performed with a Varian CP-3800 gas chromatograph, equipped with a DB-5 capillary column (30 m × 0.25 mm; coating thickness 0.25 μm) and a Varian Saturn 2000 ion trap mass detector. Analytical conditions included injector and transfer line temperatures 220 °C and 240 °C, respectively, oven temperature programmed from 60 to 240 °C at 3 °C/min, carrier gas helium at 1 ml/min, injection of 0.2 μl (10% hexane solution), and a split ratio of 1:30. Constituent identification was based on comparison of retention times with those of authentic samples, by comparing their linear retention indices (LRI) with the series of *n*-hydrocarbons and using computer matching against commercial [33] and home-made library mass spectra (built up from pure substances and components of known oils and mass spectra literature data) [33, 34].

### Contact toxicity bioassays

The two EOs were tested for contact toxicity against 7–10 day-old adults of *C. vomitoria*. Flies were treated by topical applications of the EOs with a Burkard microapplicator. A 1 ml syringe was used and 2 μl of 10, 20, 30 and, 40% EtOH solutions of the EO, corresponding to 0.2, 0.4, 0.6 and 0.8 μl EO insect^-1^ was applied on the thorax of ten unsexed adult flies. Four replicates (40 treated flies) were run for each dose. Control flies (40 each) were treated with 2 μl of ethanol. To allow the topical application of the EOs, flies were anesthetised by keeping them at -20 °C for 3 min. Insects were maintained in Plexiglas cages of 20 cm of diameter and 30 cm long (ten insects per cage) with water and sugar *ad libitum* under laboratory conditions (23 °C, 75% RH). Mortality of the flies was checked daily (every 24 h) for 4 days (96 h) and values were corrected using the Abbott formula [35].

### Fumigation toxicity bioassays

Ten unsexed adult flies were placed in an airtight glass jar (330 ml) with a screw cap. A piece of filter paper was adhered inside the cap. One hundred microliters of 10, 20, 30 and, 40% EtOH solutions of the EOs, corresponding to 30, 60, 90, and 120 μl of EO/l^-1^ of air, were applied to the filter paper. The treated filter paper was protected from direct contact with the insect by a thin layer of sterile gauze. The control jars were treated with EtOH. The jars were further sealed with Parafilm and maintained at 23 ± 1 °C, 75% RH. Each test was replicated four times and mortality was checked at 24 h.

### Oviposition deterrence

One hundred and fifty unsexed, 10–14 day-old, *C. vomitoria* adults, were placed into 75 × 75 × 115 cm cages (BugDorm-2400 Insect Rearing Tent, MegaView Science Co., Ltd., Taiwan). The flies were fed with sugar and yeast after emergence and for the whole duration of the test. Dissection and examination of a subsample of females prior to the commencement of the assays confirmed that all of them were gravid. In each cage, flies were let to lay eggs on beef meatballs (5 g) placed on Petri dishes bases (4 cm of diameter). To prevent desiccation, the meat of each meatball was mixed with 1 ml of water and 3 ml of water was poured on the bottom of the Petri dish as well. The surface of the meatballs was treated by a glass nebulizer with 100 μl of 0, 0.1, 0.5, or 1% EtOH solution of the EOs, corresponding to 0.000 (control), 0.005, 0.025, and 0.050 μl EO cm^-2^. Four meatballs, one for each treatment dose, were placed at each corner of the cage about 10 cm from the edge. Cages were collocated under fluorescent lamps, to provide even lighting (light intensity at the cages of about 14 lux), and were maintained at 23 °C and approximately 75% RH. A beaker containing 500 ml of water was positioned in each cage to maintain humidity inside the cage. The eggs laid were counted after 24 h from the beginning of the test by the piece counter function of an analytical balance. The experiment was replicated three times.

The percent effective repellence (ER%) for each concentration was calculated using the following formula [36]:

$$ \mathrm{E}\mathrm{R}\%=\left[\left(\mathrm{NT}-\mathrm{NC}\right)/\mathrm{NC}\times 100\right. $$

Oviposition Activity Index (OAI) was calculated using the formula:

$$ \mathrm{O}\mathrm{A}\mathrm{I}=\left(\mathrm{NT}-\mathrm{NC}\right)/\left(\mathrm{NT}+\mathrm{NC}\right) $$

where, NT is the total number of eggs on the treated meatball and NC is the total number of eggs on the control meatball [37].

### AChE extraction and inhibition assay

Extraction of AChE was performed as described by Seo et al. [38]. In brief, an aliquot (300 mg) of adult insects were homogenized in 4 ml of buffer (10 mM Tris-HCl, pH 8.0) containing 0.5% (v/v) Triton X-100 and 20 mM NaCl. The homogenate was centrifuged at 17,000× *g* at 4 °C for 15 min and the supernatant containing AChE was filtered through glass wool to remove excess lipid. Total protein content was quantified by the Protein Assay Kit II® (Bio-Rad) and AChE extracted was used for AChE assays.

Inhibition of AChE was determined by the colorimetric method of Ellman et al. [39] using acetylthiocholine (ATCh) as the substrate. Protein content of AChE extract was diluted to 0.1 mg ml^-1^ and the reaction mixture consisted of 500 μl of diluted AChE extract (which contained 0.05 mg protein ml^-1^) and 50 μl of EOs for each concentration (2, 5, 25, 50, 100, 125, 250 and 500 mg l^-1^ dissolved in 5% (v/v) acetone). Controls were prepared adding acetone at the same concentration and without EOs. The tube was set on incubator at 25 °C for 5 min before adding 100 μl of 0.01 M 5,5'-dithiobis-(2-nitrobenzoic acid) (DTNB; dissolved in phosphate buffer pH 7.0) and 2.4 ml of phosphate buffer (pH 8.0). Mixture was gently agitated and maintained under incubation for further 10 min at 25 °C before adding 40 μl of 75 mM ATCh (dissolved in 0.1 M phosphate buffer pH 8.0) and the mixture was then incubated for 20 min at 25 °C. The activity of AChE was measured at 25 °C from the increase of absorbance at 412 nm by a Ultrospec 2100 Pro spectrophotometer (GE Healthcare Ltd, England). Inhibition percentage of AChE activity was calculated as follows:

$$ \mathrm{AChE}\;\mathrm{inhibition}\%=\left(1-\mathrm{SAT}/\mathrm{SAC}\right)\times 100 $$

where SAT is the specific activity of the enzyme in treatment and SAC is specific activity of the enzyme in control. Residual percentage of AChE activity was calculated as (SAT/SAC) × 100. Three replicates were measured for each EOs concentration.

### Antimicrobial activity assay

The essential oils were individually tested against *Escherichia coli* ATCC 10536, *Staphylococcus aureus* ATCC BAA-1026, *Bacillus subtilis* ATCC 11774 and *Candida albicans* ATCC 10231. All the strains were purchased from the American Type of Culture Collection (ATCC, Manassan, USA) and maintained in the Laboratories of the Universidad Tecnica del Norte, Ecuador. *E. coli*, *S. aureus* and *B. subtilis* strains were grown on nutrient agar; *C. albicans* strain was grown on malt agar. The microbial strains were selected as representative of the main microbial groups agent of foodborne illnesses and other diseases of human health importance.

The antibacterial activity of EOs was determined by the agar disc diffusion method (Kirby-Bauer) as described by the Clinical and Laboratory Standards Institute (CLSI) protocol [40], with some modifications, as follows. Active microbial suspensions were made from 24-h-old agar plates using sterile saline solution until a concentration approximately 1–2 × 10^7^ UFC ml^-1^. The microbial suspension was streaked over the surface of Mueller Hinton agar (MHA, Oxoid SpA, Milano, Italy) plates using a sterile cotton swab to obtain uniform microbial growth. Under aseptic conditions, filter paper discs (diameter 6 mm, Whatman paper No.1, Oxoid) were placed on the agar plates (one disc per Petri dish to avoid any possible additive activity) and then 10 μl of each EO dilutions (corresponding to 10, 5, 2.5, 1.25, and 0.63 μl EOs per disc) was put on the discs. Control discs contained 10 μl of methanol. The inoculated plates were then incubated at 37 °C for 24 h. Microbial inhibition zones were measured using a digital calliper and expressed in millimetres (mm). Six repetitions were made for each treatment.

The minimal inhibitory concentration (MIC) and the minimal lethal concentration (MLC) were determined according to the modified procedure of Yadegarinia et al. [41] as follows: 5 ml of 10^7^ UFC ml^-1^ microbial broth were incubated in a series of tubes containing 50 μl of decreasing concentration of the oil (10, 5, 2.5, 1.25 and, 0.63 μl EO per tube). The tubes were incubated at 37 °C for 48 h under aerobic conditions and, after incubation, the growth was visually assessed. The MIC was defined as the lowest concentration of compound without visible growth. From the tubes showing no growth, 10 μl were subcultured on agar plates to determine if the inhibition was reversible or permanent. The results of the subculture were used to calculate the minimal lethal concentration (MLC). The MLC was defined as the lowest compound concentration which caused the death of 99.9% of the microbial inoculum. Three repetitions were made for each treatment.

### Statistics and data analyses

Essential oil median lethal dose (LD_50_) and median lethal concentration (LC_50_) against *C. vomitoria* adults were calculated by Log-probit regressions. Significant differences between the LD_50_ and the LC_50_ values of the two EOs were determined by estimation of confidence intervals of the relative median potency (rmp). Differences between LD_50_ and LC_50_ values were considered statistically significant when values in the 95% confidence interval of relative median potency analyses were ≠ 1.0. Effective oviposition deterrence and residual AChE activity percentage data were transformed into arcsine values before statistical analysis and processed using GLM univariate ANOVA with EO and the dose as factors. *P*-values < 0.05 were considered significant. IC_50_ values of AChE activity (inhibitory concentration needed to inhibit 50% of the enzyme activity, negative Hill slope) were calculated by nonlinear regression to a four-parameter logistic equation (variable Hill slope). Differences in sizes of inhibitory zones formed by EOs against different microbial strains were tested by Kruskal-Wallis test and means separated by Dunn-Bonferroni pairwise comparisons. Statistics were performed by SPSS 22.0 (SPSS Inc., Chicago, IL, USA) and by GraphPad Prism 5 software (GraphPad Software, San Diego, CA, USA).

## Results

### EOs extraction and GC-MS analysis

Essential oil yield (w/w) of *A. annua* was 2.25% dry weight, whereas the yield of *A. dracunculus* was 0.40%. The two EOs were pale yellow with a very aromatic, long-lasting smell.

In the *A. annua* EO, 34 constituents were identified, accounting for 96.7% of the whole oil. In the *A. dracunculus* EO, 24 constituents were identified, accounting for 99.9% of the whole oil. The principal chemical constituent of the *A. annua* EO was artemisia ketone (22.1%), followed by 1,8-cineole (18.8%), whereas methyl chavicol (73.3%) was the main chemical in the *A. dracunculus* EO. Other important volatiles were camphor (16.9%), artemisia alcohol (5.9%) and *α*-pinene (5.7%) for *A. annua* EO, and limonene and *(E)*-β-ocimene (5.4 and 5.3%, respectively) for *A. dracunculus* EO (see Table 1).

**Table 1:** Chemical composition (%) of the *Artemisia annua* and *Artemisia dracunculus* essential oils used in the assays

Constituent^a^	*LRI*	*A. annua*	*A. dracunculus*
Santolina triene	911	0.6	nd
Tricyclene	928	0.1	nd
*α*-pinene	941	5.7	2.6
Camphene	955	2.4	0.4
Sabinene	978	1.8	nd
*β*-pinene	981	1.1	3.4
Myrcene	993	2.8	0.3
Yomogi alcohol	999	1.4	nd
Pseudolimonene	1004	nd	0.3
*δ*-3-carene	1013	nd	0.3
α-terpinene	1020	0.3	0.8
*p*-cymene	1028	0.2	0.4
Limonene	1032	nd	5.4
1,8-cineole	1042	18.8	3.0
*(Z)*-β-ocimene	1043	nd	3.0
*(E)*-β-ocimene	1052	nd	5.3
*γ*-terpinene	1062	nd	0.4
Artemisia ketone	1063	22.1	0.4
*cis*-sabinene hydrate	1070	0.3	nd
Artemisia alcohol	1085	5.9	nd
Isoterpinolene	1088	nd	0.3
Terpinolene	1090	nd	1.3
*cis-p*-menth-2-en-1-ol	1123	0.2	nd
α-campholenal	1126	0.3	nd
*allo*-ocimene	1131	nd	0.2
*trans*-pinocarveol	1141	2.2	nd
*neo-allo*-ocimene	1144	nd	0.3
camphor	1145	16.9	nd
*β*-pinene oxide	1158	1.5	nd
Pinocarvone	1164	3.0	nd
*δ*-terpineol	1167	0.4	nd
4-terpineol	1179	1.2	nd
*α*-terpineol	1191	0.6	1.3
Myrtenol	1195	0.6	nd
Methyl chavicol	1197	nd	73.3
Hexyl 3-methylbutanoate	1244	0.2	nd
Isobornyl acetate	1287	nd	0.2
*α*-copaene	1377	0.2	nd
Benzyl isovalerate	1384	0.2	nd
Methyl eugenol	1403	nd	0.2
*α*-cedrene	1409	nd	0.1
*β*-caryophyllene	1419	1.8	0.1
*(E)*-β-farnesene	1459	0.1	nd
Germacrene D	1481	2.2	nd
*β*-selinene	1487	0.6	nd
Bicyclogermacrene	1495	0.5	nd
*α*-bulnesene	1507	0.2	nd
Caryophyllene oxide	1582	0.3	nd
Total identified		96.7	99.9

*Abbreviations*: *LRI* linear retention index on DB-5 column, *nd* not detected

^a^Chemical constituents ≥ 0.1%

Phenylpropanoids and monoterpene hydrocarbons (73.5 and 24.3%, respectively) represented the main chemical classes of *A. annua* EO and oxygenated monoterpenes and monoterpene hydrocarbons (75.4 and 15.0%, respectively) of *A. dracunculus* EO. For *A. annua*, another important class of chemical constituents was sesquiterpene hydrocarbons (5.6%) (Table 2).

**Table 2:** Principal chemical classes (%) in the *Artemisia annua* and *Artemisia dracunculus* essential oils used in the assays

Chemical classes	*A. annua*	*A. dracunculus*
Monoterpene hydrocarbons	15.0	24.3
Oxygenated monoterpenes	75.4	1.9
Sesquiterpene hydrocarbons	5.6	0.2
Oxygenated sesquiterpenes	0.3	0.0
Phenylpropanoids	0.0	73.5
Non-terpene derivatives	0.4	0.0
Total identified	96.7	99.9

### Oviposition deterrence

**Fig. 1:**
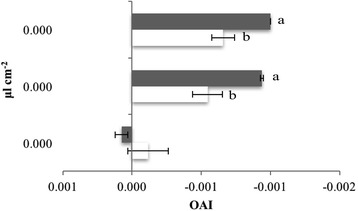
Oviposition deterrency by *Artemisia annua* and *Artemisia dracunculus* essential oils. Histograms represent the oviposition activity index (OAI) values. OAI of −0.3 and below are considered as repellents; 0.3 and above, as attractive [68]. *White bars*, *A. annua* EO; *grey bars*, *A. dracunculus* EO. Intervals in *black* represent standard errors

Both *Artemisia* EOs deterred *C. vomitoria* oviposition starting from the dose of 0.025 μl cm^-2^. At 0.050 μl cm^-2^, *A. dracunculus* EO completely inhibited *C. vomitoria* oviposition (Table 3, Fig. 1). Moreover, ANOVA showed a significantly different effect of the tested chemical on the oviposition deterrence (*F*_(1,16)_ = 7.577, *P* = 0.014) and of the dose (*F*_(3,16)_ = 16.993, *P* <0.001) with interaction effect (*F*_(3,16)_ = 5.117, *P* = 0.011). Starting from 0.025 μl cm^-2^ the *A. dracunculus* EO was more effective than EO from *A. annua* (Table 3).

**Table 3:** Oviposition deterrent effect of the *Artemisia annua* and *Artemisia dracunculus* essential oils (EOs) against *Calliphora vomitoria.* Data are presented as the mean ± standard error

	EO (μl cm^-2^)	No. of eggs laid	ER (%)
*A. annua*	0	613.67 ± 58.21 a	0.00 ± 0.00 A
	0.005	539.33 ± 399.70 ab	13.82 ± 28.27 A
	0.025	180.00 ± 180.00 bc	-69.31 ± 9.35 A
	0.050	123.00 ± 123.00 c	-78.80 ± 6.24 A
*A. dracunculus*	0	2344.67 ± 520.97 a	0.00 ± 0.00 A
	0.005	2685.67 ± 540.93 a	17.51 ± 10.62 A
	0.025	76.00 ± 76.00 b	-96.77 ± 0.63 B
	0.050	0.00 ± 0.00 b	-100.00 ± 0.00 B

*Note*: Different lower case letters indicate significant differences in total no. of eggs laid among different doses of each EO (GLM, Tukey HSD, *P* ≤ 0.05). Different upper case letters indicate significant differences in ER between the same doses of each EO (Mann-Whitney U-test, *P* ≤ 0.05)

*Abbreviation*: *ER (%)* percent effective repellence

### Adulticidal activity

*Artemisia* EOs showed good adulticidal activity, both by contact and fumigation, against the fly *C. vomitoria* even at low doses*.* By contact, EOs LD_50_ values were 0.485 to 0.786 μl per individual for *A. dracunculus* and *A. annua*, respectively. By fumigation, LC_50_ values were 49.55 to 88.09 μl l^-1^ of air for *A. dracunculus* and *A. annua*, respectively (Table 4). Relative toxicity, calculated by rmp analyses, indicated that *A. annua* EO was significantly more effective than *A. dracunculus* EO both by contact and fumigation (Table 5).

**Table 4:** Toxicity of *Artemisia annua* and *Artemisia dracunculus* essential oils (EOs) against adults of *Calliphora vomitoria* by contact and fumigation

EO	Method	LD_50_^a^/LC_50_^b^	95% CI	Slope ± SE	Intercept ± SE	*χ* ^2^ (*df*)
*A. annua*	*Contact*	0.79	0.65–1.13	3.62 ± 0.84	0.38 ± 0.25	**2.98** (2)
	*Fumigation*	88.09	75.07–107.94	10.65 ± 1.58	-20.71 ± 3.05	**5.68** (3)
*A. dracunculus*	*Contact*	0.49	0.33–0.68	5.16 ± 0.81	1.62 ± 0.27	**6.31** (3)
	*Fumigation*	49.55	44.28–54.33	6.48 ± 0.82	-10.98 ± 1.45	**3.07** (2)

*Abbreviations*: *CI* confidence interval, *df* degrees of freedom, *SE* standard error

*Note*: Values in bold indicate *P* > 0.05)

^a^Dose of EO that kills 50% of the insects treated by direct contact

^b^Concentration of EO that kills 50% of the insects treated by fumigation. Data were calculated by Probit regression analysis and expressed as μl insect^-1^ for contact tests and as μl l^-1^ air for fumigation tests

**Table 5:** Relative toxicity, calculated by relative median potency analyses (rmp), of *Artemisia annua vs Artemisia dracunculus* essential oils against adults of *Calliphora vomitoria* by contact and fumigation

Method	Estimate^a^	Lower bound	Upper bound
Contact	**1.529**	1.084	2.917
Fumigation	**1.758**	1.264	3.443

*Note*: Bold indicates significant values (95% CI ≠ 1)

^a^rmp values > 1 indicate less efficacy of *A. annua vs A. dracunculus* EO

### AChE inhibition

Both the *Artemisia* EOs inhibited the AChE of *C. vomitoria*. The AChE inhibitory activity of the two *Artemisia* EOs is summarized in Table 6. The inhibitory effect of the two *Artemisia* EOs was dose-dependent (*F*_(6,14)_ = 13.947, *P* < 0.001; *F*_(6, 14)_ = 40.738, *P* < 0.001, for *A. annua* and *A. dracunculus*, respectively). In general, EO from *A. dracunculus* was found to be a stronger inhibitor of AChE in *C. vomitoria* (IC_50_ = 202.6 mg l^-1^) compared with *A. annua* EO (IC_50_ = 472.4 mg l^-1^) (Table 7).

**Table 6:** *Artemisia annu*a and *Artemisia dracunculus* essential oils *in vitro* inhibition of acetylcholinesterase extracted from adults of *Calliphora vomitoria*. Data are expressed as the percentage of residual activity and represent the mean of three replicates ± SE

mg l^-1^	*A. annua*	*A. dracunculus*
2	96.9 ± 1.4 a	98.9 ± 1.8 a
5	95.7 ± 5.0 a	96.9 ± 5.0 a
25	98.2 ± 9.3 a	96.9 ± 4.2 a
50	84.7 ± 2.5 ab	72.4 ± 1.2 b
100	82.2 ± 2.1 ab	72.5 ± 4.3 b
125	67.9 ± 1.8 bc	59.3 ± 2.9 bc
250	54.8 ± 2.9 c	44.2 ± 1.9 c

*Note*: Different letters indicate significant differences (GLM, Tukey HSD *post-hoc* test, *P* < 0.05) within columns

**Table 7:** *Artemisia annua* and *A. dracunculus* essential oils (EOs) IC_50_ values of *Calliphora vomitoria* acetylcholinesterase (AChE) *in vitro* activity. Data are calculated by non-linear regression

EO	IC_50_	*R* ^2^	*df*
*A. annua*	472.4	0.909	20
*A. dracunculus*	202.6	0.907	21

*Abbreviations*: *IC*_*50*_ concentration (mg l^−1^) of EO that inhibits 50% of the AChE activity, *df* degrees of freedom

### Antimicrobial activity assay

The results of the antimicrobial activity of *A. annua* and *A. dracunculus* EOs revealed significant antibacterial activity whose magnitude varied depending on the EO (Kruskal-Wallis, *χ* ^2^_(1)_ = 22.485, *P* < 0.001), the microbial strain (Kruskal-Wallis, *χ* ^2^_(3)_ = 66.039, *P* < 0.001) and, the EO concentration (Kruskal-Wallis, *χ* ^2^_(4)_ = 115.945, *P* < 0.001). The diameter of inhibition zones of the tested EOs from both *Artemisia* spp. measured by disc agar diffusion method is presented in Table 8. The inhibition zone of *A. dracunculus* EO ranged from 3.5 ± 0.3 to 35.2 ± 0.6 mm for 0.63 and 10 μl disc^-1^, respectively, while *A. annua* inhibited microbial growth for a radius up to 29.3 ± 0.6 mm (10 μl disc^-1^). At 10 μl disc^-1^, the largest inhibition zones were obtained by *A. dracunculus* EO against *C. albicans* (35.2 ± 0.6 mm), while the narrowest was obtained by *A. annua* EO against *S. aureus* (7.3 ± 0.5 mm). Accordingly, MIC and MLC values showed that the most susceptible microbial pathogen was *C. albicans* (*A. annua* EO MIC and MLC ≤ 0.63 μl ml^-1^; *A. dracunculus* EO MIC and MLC ≤ 0.63 and, 1.25 μl ml^-1^, respectively) (Table 9) while, *S. aureus* was the less susceptible microorganism to *A. annua* EO (MIC = 10.0; MLC > 10.0). Overall, *A. dracunculus* EO showed the strongest and consistent inhibitory effect on microbial growth with values ≤ 0.63 μl ml^-1^ for all the microorganisms tested (Table 9).

**Table 8:** Antibacterial activity (inhibition zone, mm) of *Artemisia annua* and *Artemisia dracunculus* essential oils (EOs) against *Escherichia coli*, *Bacillus subtilis*, *Streptococcus aureus* and *Candida albicans* microbial strains. Data are presented as the mean ± standard error

EO	Dose (^a^μl.disc^-1^)	*E. coli*	*B. subtilis*	*S. aureus*	*C. albicans*
*A. annua*	10	20.8 ± 0.5 bBC	18.0 ± 0.9 bAB	7.3 ± 0.5 bA	29.3 ± 0.6 bB
	5	13.0 ± 0.5 bBC	14.3 ± 0.2 bAB	0.8 ± 0.3 bA	21.7 ± 0.6 bC
	2.5	8.7 ± 0.6 abB	8.0 ± 0.4 abB	0.0 ± 0.0 aA	17.5 ± 0.7 abB
	1.25	1.7 ± 0.2 aB	1.3 ± 0.2 aB	0.0 ± 0.0 aA	11.0 ± 0.5 aB
	0.63	0.0 ± 0.0 aA	0.0 ± 0.0 aA	0.0 ± 0.0 aA	5.3 ± 0.6 aB
*A. dracunculus*	10	15.2 ± 0.2 bA	32.0 ± 1.0b AB	14.3 ± 0.2 bA	35.2 ± 0.6 bB
	5	12.2 ± 0.4 abA	21.2 ± 0.6 abAB	11.5 ± 0.2 abA	31.2 ± 0.8 bB
	2.5	9.5 ± 0.6 abA	16.0 ± 0.4 abAB	7.3 ± 0.3 abA	28.5 ± 0.3 abB
	1.25	8.3 ± 0.3 aAB	9.0 ± 0.5 aAB	5.7 ± 0.2 aA	13.3 ± 0.6 abB
	0.63	7.8 ± 0.3 aAB	8.3 ± 0.4 aB	5.3 ± 0.3 aAB	3.5 ± 0.3 aA

*Note*: different lower case letters indicate significant differences among different doses of each EO; different capital letters indicate significant differences among microbial strains at the same doses of each EO (Kruskal-Wallis, Dunn-Bonferroni pairwise comparisons, *P* ≤ 0.05)

**Table 9:** Minimum inhibitory concentration (MIC) and minimum lethal concentration (MLC) values of the essential oils (EOs) of *Artemisia annua* and *Artemisia dracunculus* against *Escherichia coli*, *Bacillus subtilis*, *Streptococcus aureus* and *Candida albicans* microbial strains

Microorganism	*A. annua* EO		*A. dracunculus* EO	
	MIC	MLC	MIC	MLC
*E. coli*	1.25^a^	5.00	0.63	2.50
*B. subtilis*	2.50	5.00	0.63	2.50
*S. auerus*	10.00	> 10.00	0.63	2.50
*C. albicans*	0.63	0.63	0.63	1.25

^a^μl ml^-1^

## Discussion

The composition of *A. annua* and *A. dracunculus* EOs is known to be quite variable depending upon the climate, the extraction method, the plant part, the geographic location, the chemotype and, the plant genotype (for recent reviews about *A. annua* EO see [42] and regarding *A. dracunculus* EO see [43, 44]).

Chemical analyses showed quantitative and qualitative differences in the chemical composition of the two EOs. In fact, phenylpropanoids, the main chemical class of constituents of the *A. dracunculus* EO (73.5%), are completely absent in *A. annua*. On the contrary, the EO of *A. annua* was characterized by high percentages of oxygenated monoterpenes (75.4%), which constitute a minor chemical class in *A. dracunculus* (1.5%).

Essential oils have been consistently shown to be toxic and repellent against insect pests, though to date, very few studies have been conducted on their use against Calliphoridae species. In this study, both *A. annua* and *A. dracunculus* EOs, although different in chemical composition, displayed both toxic and repellent activities against *C. vomitoria*. At a dose of 0.05 μl cm^-2^ (100 μl of 1% EtOH/5 g meatball), *A. dracunculus* EO completely inhibited *C. vomitoria* oviposition. In line with our results, a complete inhibition of oviposition was previously observed for *L. cuprina* on media treated with tea tree EO [45].

The observed differences in effectiveness of the two EOs could be due to their different chemical composition. However, the complexity of the insect olfactory system makes it difficult to clarify how chemical information encoded in the repellent molecules is perceived by the insect to produce a behavioural response [46]. *Artemisia dracunculus* EO (1 ml EO in polyethylene foam wafer) has also been showed to reduce, in field conditions, the attraction of adult Japanese beetles, *Popillia japonica* Newman (Coleoptera: Scarabaeidae), to attractant-baited or non-baited traps [47] and, in an olfactometer study, *A. dracunculus* EO (2 μl of EO in 2 g of food) showed significant repellent activity against adults of the indianmeal moth, *Plodia interpunctella* [48]. Similarly, *A. annua* EO was found to be repellent against adults of *Tribolium castaneum* (Herbst) at 1% (vol/vol) concentration and above in filter paper arena test [30].

*Artemisia annua* and *A. dracunculus* EOs were also toxic by contact and fumigation against adults of *C. vomitoria*, although *A. dracunculus* EO were significantly more effective than that from *A. annua*. A different efficacy of EOs from different plants is expected although they belong to the same genera. In this case, the different bioactivity of the two EOs may be due to their very different chemical composition. For example, methyl chavicol, the main constituent (73.3%) of *A. dracunculus* EO, was absent in *A. annua* EO.

The differing efficacies of the two *Artemisia* EOs is confirmed by the 2-fold higher inhibitory effect on AChE activity exerted by *A. dracunculus* EO (IC_50_ = 202.6) as compared to that of *A. annua* (IC_50_ = 472.4). A similar inhibition of insect AChE activity has been already shown by several plant extracts [38, 49] and by some monoterpene constituents of EOs, which have indeed been recognized as the strongest inhibitors contained in EOs of different plant species [50, 51]. It has been demonstrated that the ability of monoterpenes to inhibit the AChE activity is related to their competition with the active site of the free enzyme (competitive inhibition) [51] or due to their ability to bind to either the free enzyme (but combining to a site different from the active site where the substrate binds) or the enzyme-substrate complex (mixed inhibition) [51]. In view of the above, if it was only the monoterpenoids that were suppressing AChE, one would expect a higher AChE inhibition for EO of *A. annua*, which is richer in monoterpenoids (~90%) than *A. dracunculus* (~26%). However, monoterpenoids can also be active as synergists in the inhibition of AChE [52] and thus the EO profile can be more relevant on AChE inhibition than the simple sum of their amount. In addition, it has been also demonstrated that some phenolic acids strongly inhibit the activity of AChE [51, 53, 54]. For example, López & Pascual-Villalobos [51] demonstrated that methyl chavicol, which represent about the 73% of the whole EO of *A. dracunculus*, is one of the most powerful AChE inhibitors *in vitro*. In a subsequent paper the same authors confirmed the strong AChE inhibition ability of this compound on *Sitophilus oryzae* and *Cryptolestes pusillus* [54]. The inhibitory effect of the two *Artemisia* EOs on the AChE activity suggest that the targets of their toxicity are *C. vomitoria* neuromuscular sites, the same target sites of insecticides belonging to the organophosphorus and carbamate group [55, 56]. Thus, from an applicative point of view, although EOs could represent a valid alternative to synthetic pesticides, the possibility of insurgence of cross-resistance cannot be excluded [57].

Besides the repellent and toxic effect against *C. vomitoria*, the two *Artemisia* EOs showed good antibacterial and antifungal activity except for the *A. annua* EO against *S. aureus*. Since wounds represent sites of preference for *C. vomitoria* oviposition, such antimicrobial activity can be useful in preventing secondary infections. Essential oils are lipophiles that can enter cells and interfere with the integrity and functionality of the membrane [58]. The resulting membrane permeabilisation is expected to cause loss of ions, reduction of potential, the collapse of proton pump and the depletion of ATP pool [59]. The monoterpene thymol has been shown to cause disruption of the cellular membrane, inhibition of ATPase activity, and release of intracellular ATP and other constituents [60, 61]. However, probably due to the large number of different chemical components, EOs antibacterial activity is not attributable to one specific mechanism [62] and although the antimicrobial activity of EOs is mainly due to their major components, synergistic or antagonistic effects of minor compounds should also be considered [63, 64].

Both the *Artemisia* EOs showed a strong effect against the pathogenic fungus *C. albicans.* In line with our findings, *C. albicans* was reported to be highly susceptible also to *Myrtus communis* and *Mentha piperita* EOs [41] as well as to *Origanum* spp*.* EOs [65, 66]. The action of EOs against fungi appears to be similar to those against bacteria. Tolouee et al. [67] showed that *M. chamomilla* EOs affects the permeability of *Aspergillus niger* plasma membrane causing imbalance in intracellular osmotic pressure, disruption of intracellular organelles, leakage of cytoplasmic contents and finally cell death.

## Conclusions

The prevention of pathogenic and parasitic infections is a priority for human and animal health. The efficacy of *Artemisia* EOs against the blowflies coupled with their low-cost and low-toxicity against mammals suggests that EOs could represent an alternative “soft” way to fight foodborne disease, infection, and myiasis. However, further studies are needed to establish the modality of EOs formulation and applications i.e. by microencapsulation or gel that may enable a constant release of volatiles and maximize the efficacy of the treatments.

## Abbreviations

AChE: Acetylcholinesterase
ATCC: American Type of Culture Collection
ATCh: Acetylthiocholine
ATP: Adenosintriphosphate
ATPase: Adenosintriphosphate synthase
CLSI: Clinical and Laboratory Standards Institute
EO: Essential oil
ER%: Percent effective repellence
EtOH: Ethyl alcohol
GC-EIMS: Gas chromatography-electron impact mass spectroscopy
GLM: General linear model
IC_50_: Concentration that inhibits 50% of the activity
LC_50_: Concentration that kills 50% of the insects treated
LD_50_: Dose that kills 50% of the insects treated
LRI: Linear retention index
MHA: Mueller Hinton agar
OAI: Oviposition activity
RH: Relative humidity
rmp: Relative median potency
SAC: Specific activity of the enzyme in control
SAT: Specific activity of the enzyme in treatment

## References

[1] Greenberg B (1971). Flies and diseases, vol. 1, Ecology Classification and Biotic Associations.

[2] Daniel M, Kovacova D, Roslerova V, Zuska J (1990). Synanthropic flies and other insects in the hospital area and microflora detected on the surface of their bodies. Cesk Epidemiol Mikrobiol Imunol.

[3] Graczyk TK, Knight R, Gilman RH, Cranfield MR (2001). The role of non-biting flies in the epidemiology of human infectious diseases. Microbes Infect.

[4] Pava-Ripoll M, Pearson REG, Miller AK, Ziobro GC (2012). Prevalence and relative risk of *Cronobacter* spp., *Salmonella* spp., and *Listeria monocytogenes* associated with the body surfaces and guts of individual filth flies. App Environ Microbiol.

[5] Förster M, Klimpel S, Mehlhorn H, Sievert K, Messler S, Pfeffer K. Pilot study on synanthropic flies (e.g. *Musca, Sarcophaga*, *Calliphora*, *Fannia*, *Lucilia*, *Stomoxys*) as vectors of pathogenic microorganisms. Parasitol Res. 2007;101:243–6. 10.1007/s00436-007-0522-y 17370089

[6] Förster M, Sievert K, Messler S, Klimpel S, Pfeffer K (2009). Comprehensive study on the occurrence and distribution of pathogenic microorganisms carried by synanthropic flies caught at different rural locations in Germany. J Med Entomol.

[7] Greenberg B, Kowalski JA, Klowden MJ (1970). Factors affecting the transmission of *Salmonella* by flies: natural resistance to colonization and bacterial interference. Infect Immun.

[8] Mariluis JC, Lagar MC, Bellegarde EJ (1989). Disemination of enteroparasites by Calliphoridae (Insecta, Diptera). Mem Inst Oswaldo Cruz.

[9] Fischer O, Matlova L, Dvorska L, Švástová P, Bartl J, Melicharek I (2001). Diptera as vectors of mycobacterial infections in cattle and pigs. Med Vet Entomol.

[10] Sawabe K, Hoshino K, Isawa H, Sasaki T, Kim KS, Hayashi T, et al. Blow flies were one of the possible candidates for transmission of highly pathogenic H5N1 avian influenza virus during the 2004 outbreaks in Japan. Influenza Res Treat. 2011;Article ID 652652:1–8. 10.1155/2011/652652 PMC3447300 23074659

[11] Wall R (2012). Ovine cutaneous myiasis: effects on production and control. Vet Parasitol.

[12] Millán CL, Olea MS, Juri MJD (2015). Unusual presence of *Ornidia robusta* (Diptera: Syrphidae) causing pig myiasis in Argentina. Parasitol Res.

[13] Hall MJ, Wall RL, Stevens JR (2016). Traumatic myiasis: a neglected disease in a changing world. Annu Rev Entomol.

[14] Szpila K, Pape T, Hall MJR, Madra A (2014). Morphology and identification of first instars of European and Mediterranean blowflies of forensic importance. Part III: Calliphorinae. Med Vet Entomol.

[15] Alexander JOD (1984). Cutaneous myiasis. Arthropods and Human Skin.

[16] Morris OS, Titchener RN (1997). Blowfly species composition in sheep myiasis in Scotland. Med Vet Entomol.

[17] French NP, Wall R, Cripps PJ, Morgan KL (1992). Prevalence, regional distribution and control of blowfly strike in England and Wales. Vet Rec.

[18] Baker KE, Rolfe PF, George AG, Vanhoof KJ, Kluver PF, Bailey JN (2014). Effective control of a suspected cyromazine-resistant strain of *Lucilia cuprina* using commercial spray-on formulations of cyromazine or dicyclanil. Aust Vet J.

[19] Levot GW (2012). Cyromazine resistance detected in Australian sheep blowfly. Aust Vet J.

[20] Roldán-Tapia L, Parrón T, Sánchez-Santed F (2005). Neuropsychological effects of long-term exposure to organophosphate pesticides. Neurotoxicol Teratol.

[21] Stephens R, Spurgeon A, Calvert LA, Beach J, Levy LS, Berry H, Harrington JM (1995). Neuropsycological effects of long-term exposure to organophosphates in sheep dip. Lancet.

[22] Tellam RL, Bowles VM (1997). Control of blowfly strike in sheep: current strategies and future prospects. Int J Parasitol.

[23] Littlejohn JW, Melvin MAL (1991). Sheep-dips as a source of pollution of fresh-waters, a study in Grampian Region. J Inst Water Env Man.

[24] Regnault-Roger C, Vincent C, Arnason JT (2012). Essential oils in insect control: low-risk products in a high-stakes world. Annu Rev Entomol.

[25] Isman MB (2000). Plant essential oils for pest and disease management. Crop Prot.

[26] Isman MB (2006). Botanical insecticides, deterrents, and repellents in modern agriculture and an increasingly regulated world. Annu Rev Entomol.

[27] Nerio LS, Olivero-Verbel JS, Tashenko E (2009). Repellent activity of essential oils from seven aromatics plants grown in Colombia against *Sitophilus zeamais* Motschulsky (Coleoptera). J Stored Prod Res.

[28] Bedini S, Flamini G, Girardi J, Cosci F, Conti B (2015). Not just for beer: evaluation of spent hops (*Humulus lupulus*) as a source of eco-friendly repellents for insect pests of stored foods. J Pest Science.

[29] Kordali S, Kotan R, Mavi A, Cakir A, Ala A, Yildirim A (2005). Determination of the chemical composition and antioxidant activity of the essential oil of *Artemisia dracunculus* and of the antifungal and antibacterial activities of Turkish *Artemisia absinthium*, *A. dracunculus*, *Artemisia santonicum*, and *Artemisia spicigera* essential oils. J Agr Food Chem.

[30] Tripathi AK, Prajapati V, Aggarwal KK, Khanuja SP, Kumar S (2000). Repellency and toxicity of oil from *Artemisia annua* to certain stored-product beetles. J Econ Entomol.

[31] Ujvari B, Wallman JF, Madsen T, Whelan M, Hulbert AJ (2009). Experimental studies of blowfly (*Calliphora stygia*) longevity: a little dietary fat is beneficial but too much is detrimental. Comp Biochem Phys A.

[32] Kelly MA, Zieba AP, Buttemer WA, Hulbert AJ (2013). Effect of temperature on the rate of ageing: an experimental study of the blowfly *Calliphora stygia*. PLoS One.

[33] Adams RP (2007). Identification of essential oil components by gas chromatography/mass spectroscopy.

[34] Davies NW (1990). Gas chromatographic retention indices of monoterpenes and sesquiterpenes on methyl silicon and carbowax 20 M phases. J Chromatogr.

[35] Abbot WJ (1925). A method of computing effectiveness of an insecticide. J Econ Entomol.

[36] Rajkumar S, Jebanesan A (2009). Larvicidal and oviposition activity of *Cassia obtusifolia* Linn (Family: Leguminosae) leaf extract against malarial vector, *Anopheles stephensi* Liston (Diptera: Culicidae). Parasitol Res.

[37] Cheah SX, Tay JW, Chan LK, Jaal Z (2013). Larvicidal, oviposition, and ovicidal effects of *Artemisia annua* (Asterales: Asteraceae) against *Aedes aegypti*, *Anopheles sinensis*, and *Culex quinquefasciatus* (Diptera: Culicidae). Parasitol Res.

[38] Seo SM, Jung CS, Kang J, Lee HR, Kim SW, Hyun J, Park IK (2015). Inhibitory activities of apiaceae plant essential oils and their constituents against *Aedes albopictus* and formulation development. J Agr Food Chem.

[39] Ellman G, Courtney KD, Andres V, Featerstone RM (1961). A new and rapid colorimetric determination of acetylcholinesterase activity. Biochem Pharmacol.

[40] Wayne PA (2010). Performance standards for antimicrobial susceptibility testing. Clinical and laboratory standards institute (CLSI).

[41] Yadegarinia D, Gachkar L, Rezaei MB, Taghizadeh M, Astaneh SA, Rasooli I (2006). Biochemical activities of Iranian *Mentha piperita* L. and *Myrtus communis* L. essential oils. Phytochemistry.

[42] Bilia AR, Santomauro F, Sacco C, Bergonzi MC, Donato R. Essential oil of *Artemisia annua* L.: an extraordinary component with numerous antimicrobial properties. Evidence-based complementary and alternative medicine. 2014; doi: 10.1155/2014/159819.

[43] Fraternale D, Flamini G, Ricci D (2015). Essential oil composition and antigermination activity of *Artemisia dracunculus* (Tarragon). Nat Prod Commun.

[44] Ayoughi F, Marzegar M, Sahari MA, Naghdibadi H (2010). Chemical compositions of essential oils of *Artemisia dracunculus* L. and endemic *Matricaria chamomilla* L. and an evaluation of their antioxidative effects. J Agr Sci Tech.

[45] Callander JT, James PJ (2012). Insecticidal and repellent effects of tea tree (*Melaleuca alternifolia*) oil against *Lucilia cuprina*. Vet Parasitol.

[46] Carey AF, Wang G, Su CY, Zwiebel LJ, Carlson JR (2010). Odorant reception in the malaria mosquito *Anopheles gambiae*. Nature.

[47] Youssef NN, Oliver JB, Ranger CM, Reding ME, Moyseenko JJ, Klein MJ, Pappas RS (2009). Field evaluation of essential oils for reducing attraction by the Japanese beetle (Coleoptera: Scarabaeidae). J Econ Entomol.

[48] Karahroodi ZR, Moharramipour S, Rahbarpour A (2009). Investigated repellency effect of some essential oils of 17 native medicinal plants on adults *Plodia interpunctella*. Am-Eurasian J Sustain Agric.

[49] Ryan MF, Byrne O (1988). Plant insect coevolution and inhibition of acetylcolinesterase. J Chem Ecol.

[50] Shaaya E, Rafaeli A, Ishaaya A, Neuen R, Horowitz AR (2007). Essential oils as biorational insecticides-potency and mode of action. Insecticides design using advanced technologies.

[51] López MD, Pascual-Villalobos MJ (2010). Mode of inhibition of acetylcholinesterase by monoterpenoids and implications for pest control. Ind Crop Prod.

[52] Savelev S, Okello E, Perry NSL, Wilkins RM, Perry EK (2003). Synergistic and antagonistic interactions of anticholinesterase terpenoids in *Salvia lavandulaefolia* essential oil. Pharmacol Biochem Behav.

[53] Doi S, Terasaki M, Makino M (2009). Acetylcholinesterase inhibitory activity and chemical composition of commercial essential oils. J Agr Food Chem.

[54] López MD, Pascual-Villalobos MJ (2015). Are monoterpenoids and phenylpropanoids efficient inhibitors of acetylcholinesterase from stored product insect strains?. Flavour Frag J.

[55] Matsumura F (1985). Toxicology of insecticides.

[56] Yu SJ (2008). The toxicology and biochemistry of insecticides.

[57] Lee SE, Choi WS, Lee HS, Park BS (2000). Cross-resistance of a chlorpyrifos-methyl resistant strain of *Oryzaephilus surinamensis* (Coleoptera: Cucujidae) to fumigant toxicity of essential oil extracted from *Eucalyptus globulus* and its major monoterpene, 1, 8-cineole. J Stored Prod Res.

[58] Perricone M, Arace E, Corbo MR, Sinigaglia M, Bevilacqua A. Bioactivity of essential oils: a review on their interaction with food components. Front Microbiol. 2015;6:1–7. 10.3389/fmicb.2015.00076 PMC4321600 25709605

[59] Bakkali F, Averbeck S, Averbeck D, Idaomar M (2008). Biological effects of essential oils-a review. Food Chem Toxicol.

[60] Viuda-Martos M, Ruiz Navajas Y, Zapata ES, Fernández-López J, Pérez-Álvarez JA (2010). Antioxidant activity of essential oils of five spice plants widely used in a Mediterranean diet. Flavour Frag J.

[61] Viuda-Martos M, Mohamady M, Fernández-López J, Abd ElRazik KA, Omer EA, Pérez-Alvarez JA, Sendra E (2011). In vitro antioxidant and antibacterial activities of essentials oils obtained from Egyptian aromatic plants. Food Control.

[62] Burt S. Essential oils: their antibacterial properties and potential applications in foods - a review. Int J Food Microbiol. 2004;94:223–53. 10.1016/j.ijfoodmicro.2004.03.022 15246235

[63] Daferera DJ, Basil N, Ziogas N, Polissiou MG. The effectiveness of plant essential oils on *Botrytis cinerea, Fusarium* sp. and *Clavibacter michiganensis* subsp. *michiganensis*. Crop Prot. 2003;22:39–44.

[64] Bougherra HH, Bedini S, Flamini G, Cosci F, Belhamel K, Conti B. *Pistacia **lentiscus* essential oil has repellent effect against three major insect pests of pasta. Ind Crop Prod. 2015;63:249–55.

[65] Ibrahim L, Karaky M, Ayoub P, El Ajouz N, Ibrahim S (2012). Chemical composition and antimicrobial activities of essential oil and its components from Lebanese *Origanum syriacum* L. J Essent Oil Res.

[66] Portillo‐Ruiz MC, Sánchez RAS, Ramos SV, Muñoz JVT, Nevárez‐Moorillón GV. Antifungal effect of Mexican oregano (*Lippia berlandieri* Schauer) essential oil on a wheat flour‐based Medium. J Food Sci. 2012;77:M441–5. 10.1111/j.1750-3841.2012.02821.x 22860593

[67] Tolouee M, Alinezhad S, Saberi R, Eslamifar A, Javad Zad S, Jaimand K (2010). Effect of *Matricaria chamomilla* L. flower essential oil on the growth and ultrastructure of *Aspergillus niger* van Tieghem. Int J Food Microbiol.

[68] Kramer WL, Mulla S. Oviposition attractants and repellents of mosquitoes: oviposition responses of *Culex *mosquitoes to organic infusions. Environ Entomol. 1979;8:1111–7.

